# Measuring Active Purchasing in Healthcare: Analysing Reallocations of Funds Between Providers to Evaluate Purchasing Systems Performance in the Netherlands

**DOI:** 10.34172/ijhpm.2023.7506

**Published:** 2023-09-17

**Authors:** Niek Waltherus Stadhouders, Xander Koolman, Marit A.C. Tanke, Hans Maarse, Patrick P.T. Jeurissen

**Affiliations:** ^1^Scientific Institute for Quality of Healthcare (IQ Healthcare), Radboud University Medical Center, Nijmegen, The Netherlands; ^2^School of Business and Economics, Vrije Universiteit, Amsterdam, The Netherlands; ^3^Department of Health Services Research, School for Public Health and Primary Care (Caphri), Faculty of Health, Medicine and Life Sciences, Maastricht University, Maastricht, The Netherlands

**Keywords:** Managed Competition, Purchasing, Efficiency, Hospitals, The Netherlands

## Abstract

**Background:** Purchasing systems aim to improve resource allocation in healthcare markets. The Netherlands is characterized by four different purchasing systems: managed competition in the hospital market, a non-competitive single payer system for long-term care (LTC), municipal procurement for home care and social services, and self-procurement via personal budgets. We hypothesize that managed competition and competitive payer reforms boost reallocations of provider market share by means of active purchasing, ie, redistributing funds from high-quality providers to low-quality providers.

**Methods:** We define a Market Activity Index (MAI) as the sum of funds reallocated between providers annually. Provider expenditures are extracted from provider financial statements between 2006 and 2019. We compare MAI in six healthcare sectors under four different purchasing systems, adjusting for reforms, and market entry/exit. Next, we perform in-depth analyses on the hospital market. Using multivariate linear regressions, we relate reallocations to selective contracting, provider quality, and market characteristics.

**Results:** No difference was found between reallocations in the hospital care market under managed competition and the non-competitive single payer LTC (MAI between 2% and 3%), while MAI was markedly higher under procurement by municipalities and personal budget holders (between 5% and 15%). While competitive reforms temporarily increased MAI, no structural effects were found. Relatively low hospital MAI could not be explained by market characteristics. Furthermore, the extent of selective contracting or hospital quality differences had no significant effects on reallocations of funds.

**Conclusion:** Dutch managed competition and competitive purchaser reforms had no discernible effect on reallocations of funds between providers. This casts doubt on the mechanisms advocated by managed competition and active purchasing to improve allocative efficiency.

## Background

**Key Messages**

**Implications for policy makers**

- Competitive payer system reforms, such as managed competition, do not necessarily lead to increased reallocations of funds.
- Furthermore, existing market share reallocations between hospitals did not correlate to selective contracting or quality measures.
- The market activity index (MAI) can be used to monitor budget reallocations as a proxy for efficiency gains.
- Concurrently, alternative pathways to increase efficiency should be pursued, rather than relying solely on budgetary reallocations.

**Implications for the public**

As health costs rise, ensuring that money is spend well becomes increasingly important. However, the extent that purchasers, either health insurance companies or government agencies, allocate budgets towards high-quality providers is currently unknown. Theory predicts that competitive reform stimulates efficient allocation of funds. However, comparing different purchasing systems in the Netherlands reveals little evidence of elevated allocative activity. This suggests that competitive reforms may either have limited effect on healthcare efficiency, or currently unknown mechanisms are used to improve efficiency by competitive third-party payers. Our results can guide purchasers and policy-makers to improve efforts to steer funds towards the most efficient providers.

Policy-makers increasingly seek to improve the third-party purchaser function in their aim to increase quality and reduce costs.^1^ Given the nature of consolidated healthcare markets, patient choice may not be sufficient to obtain allocative efficiency through provider competition, and third party purchasing may improve market outcomes.^2,3^ We define active purchasing (c.q. strategic purchasing, contracting, commissioning, and procurement) as interventions by third party payers to improve market outcomes. One aspect of active purchasing involves selective reallocation of resources from poor performers to best performers (supply-side steering) and channelling patients from poor to best performers (demand-side steering), eg, by patient education, consultation or prior authorization.^4-8^ Through these channels, active purchasing implies reallocation of funds towards efficient providers.^9^ We therefore expect that active purchasing induces differences in the evolution of providers’ expenditures (ie, budgets/income/revenue/market share) over time: some providers grow substantially in total expenditures, while others’ stagnate or decline. We construct a novel indicator defined as the percentage of the total market expenditure that is reallocated between providers annually. While this is a crude measure of active purchasing, it reflects a main goal of improving static and dynamic efficiency. This measure allows analysis of the effect of different institutional arrangements with respect to the third-party purchasing function.

 Literature lacks consensus on the optimal payer system to enhance provider efficiency.^10-14^ Managed competition aims to improve efficiency in the healthcare market by insurers competing over premiums, incentivizing active purchasing on the provider market. The theory of managed competition, as formulated by Alain Enthoven in 1988, assumes that third-party payers steer healthcare markets to more efficient outcomes by rewarding well-performing providers and disciplining underperforming providers.^15,16^ We use the Netherlands as case study leading in implementation of managed competition in the hospital sector.^17^ Parallel, non-competitive payer systems were retained in other healthcare markets, allowing within-country comparisons of systems with different institutional characteristics (Table 1).

**Table 1:** Overview of Sectors in Dutch Healthcare Between 2007 and 2019

**Health System**	**Hospital Care**	**LTC **	**Social Care Services**	**Personal Budgets**
Law	Health insurance Act (Zvw)	Up to 2015: Exceptional Medical Expenses Act (AWBZ). As of 2015: Long-term Care Act (WLZ)	Social Care Act (WMO^a^)	Included in AWBZ/WLZ and WMO/Zvw
Purchaser type	Competitive multi-payer system (health insurers)	Single payer system (regional care offices)	Single payer system (municipalities)	Self-purchasing
Care sectors	Hospital care, short-term mental care, primary care^b^, home nursing care (as of 2015)	Elderly care, disability care, long term mental care, home care (up to 2015)	Social support, youth care^c^, ancillary home care services (expanded in 2015)	Personal budgets for LTC and home care
Public expenses in 2019	€49 billion	€24 billion	€7 billion	€2 billion
Financing system	Premiums (set by insurer) and income-related contributions (set by government)	Payroll-related contributions (set by government)	Tax funded grant to municipalities (set by government)	Tariffs about 70% of LTC
Incentives to purchase actively	Yes, budgetary gains may result in competitive advantage through lower premiums	No, budgetary gains of active purchasing flow back to the government	Yes, budgetary gains can be used to fund other municipal projects	Yes, budgetary gains accrue to the patient
Financial risk of payer	Increased over time through reduced ex-post equalization	Absent (overspending is forbidden)	High (no ex-post equalization)	High (personal two-way risk)

Abbreviations: LTC, long-term care; Zvm, Zorgverzekeringswet; AWBZ, Algemene wet bijzondere ziektekosten; WLZ, Wet Langdurige Zorg; WMO, Wet maatschappeljke ondersteuning. ^a^ WMO was expanded in 2015 (WMO-2015). ^b^ Primary care includes family physician care, dental care, physiotherapy, pharmaceuticals and paramedical treatments. Primary care is not included in our dataset. ^c^ Youth care includes specialty (mental) care for children and parents, and is excluded from the dataset.

###  Hospital Care

 The hospital sector comprises 8 University Medical Centers (UMCs), about 100 general hospitals and about 300 independent treatment centers (ITCs). Some hospitals have multiple locations, and some ITCs are chain-affiliated. All hospitals are private entities, and predominantly non-profit foundations as a legislative ban on profits applies, although some ITCs are commercially oriented.^18^ A system of managed competition was implemented in the hospital sector in 2006, which included, among others, competition between insurers, room for free-pricing and selective contracting and the liberalization of the certificate-of-need regulations.^18-20^ Currently 10 insurance companies compete in the market, with four insurance companies (the ‘big four’) possessing about 90% of the market. Insurers aim to set low premiums to be competitive. As the theory of managed competition postulates, active purchasing allow insurers to save costs and reduce premiums. The percentage of consumers that switch between insurers varies around 7% each year.^21,22^ Financial risk of insurers has substantially increased in the research period as a consequence of the government’s policy to restrict ex-post equalization.^20^ As the market is designed as a competitive market, we expect that payer incentives for active purchasing are high, leading to substantial reallocations of expenditures towards efficient providers.^19^

###  Long-term Care

 The long-term care (LTC) sector, including elderly care, disability care, long-term mental care, and, up to 2015, home care, is divided into 32 regions, where a non-profit regional care office acts as single payer.^23^ All +-750 providers are private entities, sometimes chain-affiliated or having multiple locations. As in hospital care, a legislative ban on profits applies, although some commercially oriented providers remain. Contrary to hospital care, incentives for active purchasing are (largely) absent in LTC. Each regional care office receives a block grant from the government for contracting providers. Since offices cannot overspend or build reserves, they do not bear any financial risk (with the exception of administrative costs), although underspending may result in block grand reallocation. This ‘threat’ incentivises each regional office to spend its full budget.^24^ Our hypothesis is that a single payer system without financial risk displays low active purchasing, and as a result, few budget reallocations.

###  Social Care Services

 The introduction of the Social Support Act in 2007 delegated social care procurement from the LTC system to municipalities.^25^ In 2015, supportive home care was transferred from LTC to municipalities. As both the introduction in 2007 and the addendum in 2015 went along with a significant budgetary cut, municipalities are expected to have strong incentives for active purchasing.^26^ WMO (Wet maatschappeljke ondersteuning) services are procured by individual municipalities or small groups of collaborating municipalities through competitive tenders. About a third of WMO is provided by large specialised companies (eg, cleaning companies), about a third by independent contractors, and about a third by LTC-providers as secondary source of income. Only the latter is formally non-profit, other providers may have profit motives. In purchasing, municipalities are financially at risk. If municipalities spend more than the annual state grant, budgetary cuts are required, as municipalities have limited ability to raise additional resources.^27^ Concurrently, public spending may be increased on other municipal expenditures in case of underspending on social support. Our hypothesis is that the Social Support Act contains strong incentives for municipalities to engage in active purchasing and reallocating budgets towards efficient providers.^25^

###  Personal Budgets

 Lastly, patients may self-procure social services and LTC through personal budgets. Patients that meet requirements for receiving home care and in-kind LTC have the option to apply for a personal budget of about 70%-80% of in-kind tariffs to self-procure care. The personal budget system is characterized by the absence of a third-party payer (insurer, care office, and municipality). Due to the nature of the opt-out system, personal budget holders are likely to critically assess the quality of providers. Furthermore, personal budget holders have a financial motive to be selective because the budget they receive is less than in-kind LTC-tariffs. Therefore, we expect that budget holders will engage in active purchasing by selecting efficient providers.

###  Measuring Purchaser Activity

 We compare purchasing activity in different payer systems in the Netherlands through its effect on changes in provider expenditures. We research six provider markets with different institutional arrangements: Hospital care (competitive multi-payer system), long-term elderly care and long-term disability care (both non-competitive single payer system), mental care (mixed), social care services (municipal purchasing), and personal budgets (self-purchasing). Due to complexities of distinguishing short-term mental care (Zvw, Zorgverzekeringswet) and long-term mental care (Algemene wet bijzondere ziektekosten/Wet Langdurige Zorg, AWBZ/WLZ) in the data, mental care is included as a mixed purchasing system. As the theory of managed competition hinges on active purchasing as a means of achieving efficiency, we hypothesize that managed competition increases reallocation of funds between hospitals. As many factors besides active purchasing may affect changes in hospitals’ income, we test which institutional and provider characteristics are related to the rate of reallocation in the hospital market. Interestingly, two major competitive reforms occurred during our study period, allowing comparison of institutional changes within a single market. In 2007 the hospital sector was gradually reformed to a competitive multi-payer system. In 2015, LTC was reformed, including reallocation of home care from a regional single payer system partly to municipal payers (ancillary care) and partly to the managed care system (home nursing care). We hypothesize that these competitive reforms stimulated active purchasing, increasing provider budget reallocations.

## Methods

###  Data Collection

 To measure changes in providers’ expenditures over time, annual statements were collected for all Dutch health providers from 2007 to 2019, using the online dataset DigiMV.^28^ Annual statements contain provider expenditures specified to payer system and type of care. Legislation mandates all providers of hospital care, mental care, home care and LTC to publicly disclose annual statements, although some exceptions apply (eg, very small companies and independent medical specialist associations). As annual statements contain information on the current year as well as the previous year, data on 2006 was retrieved from the 2007 annual statements. Data were corrected for input errors and missing data by crosschecking with financial reports from the provider website. By internet searching providers with missing years in the dataset, bankruptcies, mergers and takeovers were identified. Data from merged providers were aggregated retrospectively starting from 2006 to clean up administrative reallocation effects of mergers. Within a parent company, no expenditures per location, chain member or subcontractor are provided, allowing analysis at the level of parent company only.

 The final dataset contains 3066 providers. Of those, 685 providers, representing 92% of overall expenditures in the dataset, have data for at least 13 consecutive years. Predominantly small providers are more likely to emerge during the study period, experience bankruptcy, or have missing data for multiple years (eg, due to the voluntary nature of depositing annual statements). Combining the hospital and LTC sector, 1396 providers entered the market in the research period, responsible for 4.3% of total expenditures. In the same period, 206 providers exited the market, (1.8% of expenditures). 743 providers first entered and then exited the market during the study period (0.5% of total expenditures). The number of small providers that are exempt from annual statement provision is unknown.

 To express the coverage of the dataset in terms of total expenditures (data completeness), total sector spending is derived from official government financial statements (2006-2022). The most recent expenditure data for a given year is used. Mean completeness is 93% for hospital care, 91% for mental care and 95% for elderly and disability care. Completeness of the data for social care (73%) and personal budgets (14%) is significantly less, because the mandate to publish annual statements does not apply for companies that exclusively provide social care and personal budget care. In all sectors, distribution of funds is highly unequal with the 20 biggest providers (C20) capturing 43%, 22%, 47%, 65%, 21%, and 7% of the 2016 market for hospital care, elderly care, disability care, mental care, municipal care and personal budgets, respectively.

###  Constructing the Market Activity Index

 We define the market activity index (MAI) as the part of total sector expenses that is reallocated between providers between years. In each year, market share (*MS*) for provider *i* is calculated by dividing provider expenditure *E* in sector *k* by total expenditures *S* in sector *k*:


(1) $$MS_{i,t}^{k}=E_{i,t}^{k}/S_{t}^{k}$$

Where sector *k* is hospital care, elderly care, disability care, mental care, municipal care and personal budgets, and *t* is ‎ between 2006-2019, with t(0) = 2006. As denominator we use total expenditures according to government statements, which is less sensitive to changes in dataset composition. As robustness check, we use the sum of expenditures in the dataset as denominator. To calculate the MAI, we sum the absolute change in market share for each provider *i* in sector *k*, ranging from 1 to *I*:‎


(2) $$MAI_{t}^{k}=\frac{1}{2}\sum_{1}^{I}\left|MS_{i,t}^{k}-MS_{i,t-1}^{k}\right|$$

We divide the sum by two, as a gain in market share for one provider by definition means an equivalent loss in market share for other providers^[1]^. Because very small providers are missing from the dataset, the sum of market shares of providers in the dataset does not add up to unity. Therefore, we extrapolate the MAI to total sector expenditures by assuming that reallocations in unobserved market shares of small providers are equal to observed share reallocation fractions:


(3) $${\hat{MAI}}_{t}^{k}=\frac{MAI_{t}}{\sum_{1}^{I}MS_{i,t}^{k}}$$

To discern between annual fluctuations (eg, increases in one year and reductions in the next) and structural reallocations (eg, a trend of changes in the same direction for n years in a row), we calculate structural reallocations as market share changes between a period of n years:


(4) $$MAI_{t}^{k}=\frac{1}{2n}\sum_{1}^{I}\left|MS_{i,t}^{k}-MS_{i,t-n}^{k}\right|$$

Mean structural reallocations are by definition equal or lower than mean annual market share reallocations. For example, the total market share change between 2011 and 2015 is by definition equal or less than the sum of each absolute annual change over the same period. Although MAI is an imperfect indicator of active purchasing, it has a number of advantages over existing indicators (Box 1).

**Box 1. A Closer Examination of the Market Activity Index**

The MAI is a measure of market stability: a low MAI signals that providers have relatively stable shares in the market over time. Contrary, high MAI signals volatility. The optimal MAI is uncertain: it is nonzero, as reallocations towards efficient providers is desired. Furthermore, markets need to respond to trends in consumer needs, demographics and innovation, which increases MAI when providers are impacted heterogeneously. However, volatile markets may have high costs of acquiring capital and lost investments due to bankruptcies. The optimal MAI is likely dependent on market characteristics, which means that each market has a unique optimum, which may change over time. For example, emerging markets tend to be highly volatile, while mature markets may me more stable. High volatility is expected in markets with low fixed investments and low cost of entry and exit.

External trends may further impact MAI. For example, temporary losses of provider income (eg, due to renovations) unambiguously increase MAI. Mergers and acquisitions have a major impact MAI, as acquired providers transfer their full market shares to the new parent company. Therefore, we correct for mergers retrospectively to account for these administrative changes. This does allow for real effects of mergers, eg, due to increased market power of the merged company or due to concentration of specialized care. These real effects may be considered as part of market activity potentially impacted by active purchasing and are included in the MAI. Bankruptcies, whether or not the result of active purchasing, unambiguously increase MAI. Lastly, policy changes may impact MAI. These may be administrative changes in funding, for example when certain services are transferred from one sector to another. If these services are distributed heterogeneously between providers, MAI is expected to increase due to denominator effects. Real policy changes, eg, investments in quality or cost containment, may increase MAI if effects are distributed heterogeneously over providers. While there have been minor policy changes during the study period that predominantly impact sectoral spending [1], they likely have a minor impact on the MAI. It is difficult to correct for these contributing factors, implying that the MAI overestimates reallocations resulting from active third party purchasing.

The relation between competitiveness and reallocation of funds is ambiguous. In equilibrium, perfectly competitive markets may exhibit low reallocations. However, small perturbations may induce large changes in allocations. Reallocations may be expected to decline in more concentrated markets if market power is exerted by providers to retain market shares. Traditional market indicators, such as the Hirschman-Herfindahl index and comparable concentration indices carry the implicit assumption that more competitive, ie, less concentrated markets perform better. However, allocative changes due to active purchasing generally have little effects on concentration indices, and may even leave them unaffected, for example when funds are reallocated between providers without affecting the overall distribution of funds. Conversely, mergers may impact concentration indices without affecting real budgetary allocations. The assumption that less concentrated markets function better may not hold in regulated markets, where concentration and reduced competitivity are reasons to install active third-party purchasers in the first place. This calls for additional indicators that measure actual market performance, of which reallocations of funds between providers is one factor. However, other indicators may be needed; third parties may engage in strategic purchasing that leave the MAI unaffected. Examples include uniform across-the-board quality improvements or cost containment, as well as activities that aim to improve provider efficiency within a fixed budget. So while MAI is one indicator of active third party purchasing, it may not fully capture all potential outcomes of active purchasing.

----------------------

Abbreviation: MAI, market activity index.

^a^ For example, short term recovery for elderly (€0.7 billion) was transferred from LTC to hospital care in 2013.

###  Trend Analysis and Explanatory Regression Analysis

 To assess the effect of managed competition on MAI, we compare the hospital MAI over time and to other sectors. We hypothesize that the incentives for active purchasing are reflected in the height of the MAI, placing the hospital sector on par with municipal care and personal budgets. As robustness checks, we exclude the effect of entry and exit on the MAI. Next, we analyse the effect of competitive reform on MAI. In 2015, home care was reallocated from the LTC-sector to the managed competition health insurance sector (home nursing care) and municipalities (home ancillary care). While these reallocations cannot be distinguished in the data directly, we can indirectly gauge the effects by focusing on a subset of providers that specialize in home care (over 90% of clients receive home care). We analyzed MAI before and after the reform, to research whether an institutional setting aimed to promote active purchasing results in higher MAI within the home care sector.

 Next, we performed additional regression analyses on an individual provider level to further investigate active purchasing and its relation to market characteristics. We use two outcome measures at the provider level that relate directly to the MAI: (1) Relative changes in hospital market share over time, reflecting causal impact on market share growth (or decline); and (2) Absolute changes in hospital market share over time, reflecting impact on the size of market share reallocations (either positive or negative). We use the extent of selective contracting as proxy for active purchasing, use quality measures as a proxy for efficiency, and we correct for provider size, type of hospital, and fixed assets as a measure of flexibility.

 Selective contracting is an important tool for active purchasing under managed competition to increase efficiency.^15,29^ Data on the extent of selective contracting between 2015 and 2019 is obtained from Mediquest, a company that constructs online tools for consumers to facilitate insurance plan choice. For each insurer plan, data is collected on whether each hospital is contracted, but also within each contracted hospital is registered whether certain procedures are contracted. It may be the case that a hospital is contracted in an insurer plan, but specific treatments are excluded. In total 1655 procedures are eligible for selective contracting (for example, elective breast cancer care). However, not all hospitals offer all procedures. On average, hospitals and ITCs provide 475, respectively 37 eligible procedures. We construct a contracting index defined as the fraction of offered procedures contracted by an insurance plan. The contracting index is zero when a hospital is not contracted, one if all procedures are contracted, and between zero and one if certain procedures are excluded. Next, an average contracting index (*ACI*), ‎weighed by the market share of each insurer, is calculated for each provider, ranging from zero (not contracted for any offered procedure in any insurer plan) to one (contracted for all offered procedures in all insurer plans)^[2]^. For 161 providers, data on all years is available, covering 88% of total hospital expenditures. All hospitals and ITCs experience selective contracting to a certain extent, with mean contracting indices of 0.86 and 0.56 for hospitals and ITCs, respectively. We hypothesize that when an insurer excludes a provider from his network, the total expenditures (ie, market share) of that provider are reduced. Vice versa, if an insurer adds a provider to the plan network, provider income (ie, market share) is expected to increase. As robustness check, we differentiate our analysis between hospitals, UMCs, and ITC.

 Provider size may affect the potential of payers to reallocate funds. Furthermore, provider concentration increased during our study period, particularly in the hospital sector. On the one hand, provider market power and limited alternatives may reduce the potential to reallocate budgets between providers. On the other hand, large providers may have more flexibility to accommodate large reallocations in absolute terms, ie, a reallocation of one million euro is easier to accommodate for large providers than for small providers. Literature on provider concentration finds mixed results.^30-33^ Depending on which effect dominates, provider concentration could result in differences in the baseline level of MAI between sectors. Therefore, we research the relation between reallocations and provider size in the previous year, both in absolute terms and in relative terms. Quadratic effects are estimated for provider size to allow nonlinear effect. As robustness checks, we repeat the analysis for the other sectors.

 The potential for reallocation of funds may also relate to care characteristics, eg, patient length-of-stay. Inpatient care, having longer lengths of stay, may have intrinsically higher MAI. Therefore, we differentiate between traditional hospitals (mostly in-patient care) and ITCs (mostly outpatient care). As a robustness check, we repeat the analysis for LTC, distinguishing between nursing home care (in-patient residential care) and home care (out-patient non-residential care). Related, sectors with high fixed costs may have lower baseline MAI, because high fixed costs limits the capacity of providers to cope with large negative income shocks. Fixed costs (*FC*) are defined as costs related to real estate: interest payments, rents and capital depreciation as percentage of total expenditures. As a robustness check, we estimate type and fixed costs separately to avoid potential multicollinearity.

 The optimal MAI may be a trade-off between budget continuity and allocative efficiency. If funds are redirected towards high-quality providers, efficiency may still be achieved. To test this, we employ a method similar to Chandra et al, relating market share changes to quality performance measures in US regional markets.^9^ While our data lacks regional market indicators, this is less problematic in a small country as the Netherlands, which could be regarded as a single market. As quality measures, we calculate mean z-scores of publicly available structure, process and outcome indicators from 2010-2018. For details on Dutch hospital quality indicators, see Wackers et al.^34^ As robustness check, — following Chandra et al — we estimate a pooled regression relating absolute size to quality measures to correct for the possibility that large providers have higher quality overall. Due to limited data availability of hospital standardized mortality ratio (HSMR) data (available for general hospitals and UMCs only from 2010-2013 and 2016-2019), we include HSMR in a robustness check.

###  Regression Analysis

 We estimate two sets of multivariate pooled linear regressions:


(5) $$\Delta m_{i,t}=\beta_{0}+\beta_{1}\Delta ACI_{i,t}+\beta_{2}S_{i,t-1}+\beta_{3}S_{i,t-1}^{2}+\beta_{4}FC_{i,t-1}+\beta_{5}ICT_{i}+\beta Q_{i,t-1}+\beta t+u_{i,t}$$

(6) $$\left|\Delta m_{i,t}\right|=\beta_{0}+\beta_{1}\left|\Delta ACI_{i,t}\right|+\beta_{2}S_{i,t-1}+\beta_{3}S_{i,t-1}^{2}+\beta_{4}FC_{i,t-1}+\beta_{5}Q_{i,t-1}+\beta_{6}ICT_{i}+\beta t+u_{i,t}$$

*Δm*_i,t_ is the change in market share of provider *i*between year *t* and year *t-1*, while |*Δm*_i,t_| is the absolute change in market share of provider *i* between year *t* and year *t-1*. The first regression can be interpreted as testing causal relations between confounders and the *direction* of market share changes, while the second regression tests how confounders affect the *absolute size* of market share changes, irrespective of the direction.

 As confounders, *ΔACI*_i,t_ is the change in ACI between year t and year t-1 for provider *i*, *S*_i,t-1_ is the size of provider *i* in year *t-1*, *FC* are fixed costs as percentage of provider expenditures, *ICT* is one if a provider is an ITC and zero if the provider is a regular hospital or UMC, ***Q***_i,t-1_ is a set of quality measures (HSMR, structure, process, outcome), and *t* are year dummies. The error term *u*_it_ is corrected for panel clusters. The sample is limited by contracting index data (from 2015) and HSMR (hospitals only from 2010). To address missing (at random) values for variables *ACI*, *FC*, and *Q*, ‎we perform multiple imputation using a multivariate normal model and a Markov chain Monte Carlo procedure, with ten iterations and size and (relative) change in market share as predictors. As additional robustness checks, regressions excluding ACI and HSMR are run. Regressions are estimated using Stata 17.

## Results

###  Descriptive Statistics

 Descriptive statistics of total sector expenditures and spending growth are given in Supplementary file 1 (Tables S1 and S2). Growth in total spending is irregular in all sectors. For example, the hospital budget increased by 11% in 2013, while nominal growth was only 3% in 2011.^35^ Similarly, LTC grew by 11% in 2012 and by 0% in 2013.^35^ Municipal spending doubles in 2015 due to the reform reallocating home care partly from LTC to municipalities. Hospitals have higher mean expenditures than other sectors, but variation in provider budgets is large in all sectors. In the hospital sector, 49% of providers are ITCs, while for elderly care and disability care providers, 69%, respectively 45% of patients are receiving home care (out-patient setting). Capital expenditures on average comprises 10% of total expenditures.

 Table S3 displays provider level statistics for the hospital market. The sample covers 90% of the hospital market. Average market share is 0.26%, and the maximum provider market share is 2.4%. The mean change in market share between two years is 0.01%, with a maximum change of 0.74%. The ACI is 0.77, which means that the average provider of hospital care is contracted for 77% of all available types of treatments over all insurance companies. The mean change in contracting index between two years is 2.2%. The mean fixed costs in the sample are 7%, which is lower than in other sectors.

###  Trend Analysis: Market Activity Per Sector

**Figure 1:**
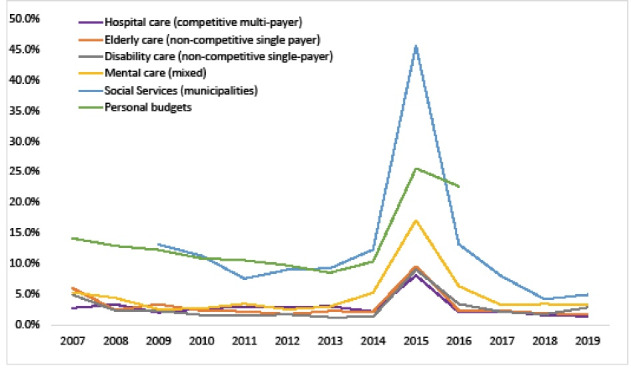
Market Activity Index Over Time for Dutch Healthcare Sectors, 2006/2007-2018/2019.

 Hospital MAI was between 2% and 3% in non-reform years, similar to LTC (Figure 1). Contrary, MAI in social services and personal budgets was between 9%-13%. Generally, MAI is lower than changes in total sector expenses, suggesting that fluctuations in the macro budget are predominantly passed on to providers with little concern for reallocations. Given all policies taken to improve the purchasing function in hospital care, specifically reductions in ex-post compensations, market reallocations in the hospital sector were expected to increase over time. However, Figure 1 shows a downward trend in MAI in all sectors. In the hospital sector, MAI on average is lower after 2015 (1.8%) than before 2015 (2.7%).

 Peaks in MAI in 2015 predominantly reflect administrative changes induced by reforms. In the hospital sector, payment system reform incorporated independent medical specialist payments into hospital expenses of most general hospitals in 2015, causing administrative changes in market share distributions. In the other sectors, reallocation of funds from LTC to municipalities and health insurers led to large reductions in LTC market share and large increases in municipal market share for those most affected by the change.^36^ To – partly – correct for administrative effects, we aggregate LTC-provider expenditures over all sectors (Figure S1 in Supplementary file 1). After reform, real MAI increases of 1%-2% are found, potentially induced by the reform. Comparable total and public MAI indicates limited substitution between public and private provider income.

 The 2015 reform does allow assessment of MAI of home care providers under different payer systems (Figure S2). After 2015, MAI for home care purchased by health insurers slightly increased in the year after reform before returning to pre-reform levels (Figure S3). Contrary, increases are much higher for home care procured by municipalities. In both sectors, MAI is initially higher in the years after reform but declines over time.

**Figure 2:**
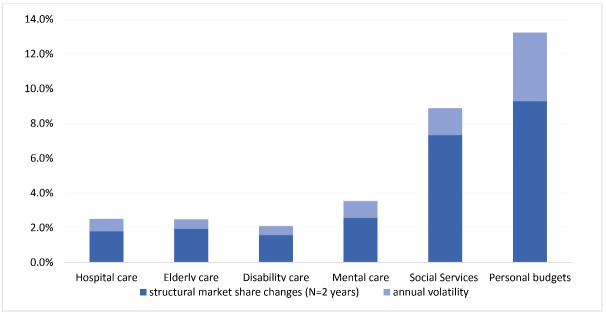
Mean Market Activity Index for Dutch Healthcare sectors in Non-reform Years, 2007/2008-2018/2019.

Figure 2 disaggregates annual and structural reallocations. Most of the MAI is structural, ie, a two-year trend in increases or declines in expenditures, suggesting year-to-year fluctuations only explain a small part of sector reallocations. When correcting for year-to-year fluctuations, structural MAI in the hospital sector declines to 1.8% versus 1.9% in elderly care and 1.6% in disability care, suggesting structural reallocations are similar under competitive multi-payers and non-competitive single payers. Overall, the trends suggest that a system of managed competition does not necessarily result in higher reallocations of funds.

 As robustness check, we recalculated MAI excluding providers that entered or exited the marked during our study period. Entry and exit are responsible for about 0.2 percentage point change in the budget annually in both the hospital sector and LTC sector (Figure S4). Furthermore, we find similar results when recalculating MAI using non-missing providers only (Figure S5).

 Overall, MAI in hospital care appears relatively low given the institutional emphasis on active purchasing. However, certain characteristics of the hospital market may lead to inherently lower reallocations, irrespective of active purchasing. In the following section, we assess the effect of market and provider characteristics on the MAI to check the robustness of our findings.

###  Regression Analysis Results

Table 2 displays the results of the main regressions. No significant relation was found between changes in selective contracting and changes in provider income, both in relative terms and in absolute terms. This finding is robust to the imputation procedure (Table S4). Robustness checks (Table S5) find a positive correlation between direction of market share growth and contracting index for ITCs, but not for hospitals or UMCs. No effects on absolute size of reallocations were found, suggesting contracting may predominantly affect small ITCs. Size does increase reallocations in absolute terms, with larger provider experiencing larger absolute changes in market share. Provider size is negatively correlated to market share growth, indicating that small providers grow at the cost of large providers, but with diminishing returns to size. To validate these findings, we repeat the analysis for all years and for all sectors (Tables S5-S8). In all sectors, larger providers experience larger absolute changes in budget growth. The effect of size on relative market share changes is ambiguous; in the full sample the signs flip in the hospital market, and in other sectors no clear relation emerges. While the effect of provider size on the *direction* of reallocations is ambiguous, the *size *of reallocations increases with provider size in all health markets. This indicates that concentrated markets may demonstrate larger absolute changes in budgetary allocations and therefore larger MAI. The main regression displays a significant effect of fixed costs on absolute size of reallocations, suggesting that high fixed costs are associated with smaller reallocations of funds. Sensitivity analyses (Table S5) finds similar results for elderly care and personal budgets, but not for other sectors, suggesting the results are not robust over specifications and sectors.

**Table 2:** Main Regression Results (Pooled Linear Regression With Clustered Standard Errors And Year Fixed-Effects)

	**Relative Market Share Changes**	**Absolute Market Share Changes**	**Relative Market Share Changes, Including HSMR**	**Absolute Market Share Changes, Including HSMR**
Delta ACI	‎-0.04 (0.04) ‎	‎-0.04 (0.06)‎	‎-0.03 (0.06) ‎	‎0.03 (0.05)‎
MS (t-1)	‎-1.76* (0.84) ‎	‎4.00** (1.45)‎	‎0.70 (1.02) ‎	‎2.84*** (0.77)‎
MS^2^(t-1)	‎35.47* (15.97) ‎	‎-50.99 (32.91)‎	‎-15.31 (26.18) ‎	‎-34.90* (14.11)‎
FC (t-1)	‎0.02 (0.02) ‎	‎-0.03* (0.01)‎	‎-0.05 (0.15) ‎	‎-0.124 (0.12)‎
ITC dummy	‎0.02 (0.01) ‎	‎0.03 (0.03)‎	‎#‎	‎#‎
Structural Q (t-1)	‎-0.002 (0.01) ‎	‎-0.01 (0.01)‎	‎-0.03 (0.02) ‎	‎-0.03 (0.02)‎
Process Q (t-1)	‎-0.005 (0.01) ‎	‎0.01 (0.01)‎	‎-0.01 (0.01) ‎	‎0.01 (0.01)‎
Outcome Q (t-1)	‎0.002 (0.01) ‎	‎0.01 (0.01)‎	‎0.02 (0.01) ‎	‎0.01 (0.02)‎
HSMR (t-1)			‎-0.03 (0.02) ‎	‎-0.02 (0.01)‎
Year = 2016	Baseline			
Year = 2017	‎0.01 (0.01) ‎	‎-0.01 (0.01)‎	Baseline	
Year = 2018	‎0.03** (0.01) ‎	‎-0.01 (0.01)‎	‎0.02* (0.01) ‎	‎-0.001 (0.005)‎
Year = 2019	‎0.03** (0.01) ‎	‎-0.01 (0.01)‎	‎0.02*** (0.01) ‎	‎-0.004 (0.005)‎
Constant	‎-0.01* (0.01) ‎	‎-0.001 (0.01)‎	‎0.01 (0.02) ‎	‎0.03 (0.02)‎
# Observations	‎285‎	‎285‎	‎185‎	‎185‎
F-statistic	‎4.6‎	‎6.8‎	‎3.7‎	‎4.5‎
R^2^	‎0.08‎	‎0.16‎	‎0.11‎	‎0.18‎

Abbreviations: ACI, average contracting index; MS, market share; HSMR, hospital standardized mortality ratio; ITC, independent treatment center; FC, Fixed costs. # Omit-ted due to collinearity (all observations of HSMR have ITC = 0). Standard errors are in parentheses. * 5% significance; ** 1% significance; *** 0.1% significance.

 After correcting for size (and fixed costs), no significant differences in reallocations are found between ITC and hospitals. While outpatient care displays higher reallocations (Figure S6), this can be fully explained by differences in size. Robustness checks (Tables S7 and S8) find that for elderly care and disability care, outpatient care is associated with lower budgetary growth. Outpatient elderly care displays larger reallocations than inpatient elderly care. Quality measures do not have any significant effect on market share changes. This is validated in the robustness checks (Table S9). Interestingly, process, outcome and HSMR are negatively associated with size, but do not affect changes in market share. Larger providers do have higher structural quality, but higher structural quality is associated with lower market share growth in the following year. This effect is not robust to restriction to the subset of hospitals with contracting data, however.

## Discussion

 Active purchasing is advocated to improve healthcare efficiency. While active purchasing may take different forms, one major tool to improve efficiency is reallocation of funds from underperforming providers to well-performing providers. We designed and applied the MAI to measure reallocations of funds between providers in Dutch health markets under different institutional settings. The municipal local procurement system and procurement through personal budgets displayed high MAI, consistent with previous findings.^36^ Contrary to our hypothesis, competitive managed competition in the hospital care sector did not display higher MAI than the non-competitive single payer LTC sector. This suggests that managed competition in the health insurance market did not increase reallocations of funds. Although the extent of reallocations is only one indicator of active purchasing, the results suggest that managed competition in the Netherlands does not perform as predicted by theory.

 Additional analyses confirm this conclusion. First, no correlation was found between the extent of selective contracting and budget reallocations, suggesting that selective contracting is not used to reallocate market shares. Second, no correlation was found between budget reallocations and quality measures, suggesting that any reallocations are not the result of quality gradients, as would be expected when active purchasing works as intended. Third, low MAI cannot be explained by market characteristics. We found that while provider size has ambiguous effects on the direction of market share changes, the size of reallocations is unambiguously larger for large providers. This is unsurprising: large providers may be able to accommodate larger changes in absolute euro amounts (eg, a one million euro change in provider income is more easy to accommodate for a provider with one billion euro in revenue than for a provider with 20 million euro in revenue). Since the size of reallocations are positively correlated to provider size, a more highly concentrated hospital market would allow higher base MAI than less concentrated sectors, such as elderly care. We also found some indications that high fixed costs reduce the size of reallocations. As fixed costs are lower in the hospital sector than in LTC, a higher baseline MAI could be expected in hospital care. The proportion of outpatient care has no effect on MAI, after correcting for size and fixed costs, suggesting lower percentages of outpatient care in the hospital sector has limited influence on baseline MAI. To conclude, we found no evidence for hospital market characteristics biasing MAI downwards; actually we would expect higher baseline MAI in the hospital sector, which confirm the finding that reallocation of funds between providers is relatively low in the hospital sector.

 These results quantitatively validate international qualitative experiences on strategic purchasing, both for the Dutch managed care system as for active purchasing in other countries.^8,17,37^ Second, our results confirm international comparisons finding few structural differences between payer systems.^10-14^ Third, our results underline that competitive reform may not increase allocative efficiency. In the United Kingdom, for example, waves of competitive purchaser reforms had little effect on provider practice.^10,38-41^

 Our study finds preliminary evidence that allocative activity is correlated not with the number of payers or payer competitiveness, but with decentralized purchasing and purchaser’ monetary incentives. International studies on the effect of decentralized purchasing on budgetary reallocations (eg, in Scandinavian systems) could validate these preliminary results.

 Interestingly, we found no relation between reallocations and quality measures, although the validity of quality indicators that are publicly available for the Netherlands may be questionable.^42^ Furthermore, literature on the effects of quality differences on prices, volumes or market share, displays mixed results,^9,43-46^ although for specific treatments a positive relationship between quality and market share -in terms of patient numbers- has been found.^9,47-49^ One potential explanation for these conflicting findings is that growth in the number of new patients may not correlate with growth in hospital expenditures, for example when prices or treatment intensity decline correspondingly.^50^ Furthermore, it remains unclear whether active purchasing or patient choice drives these results.^43,50^ Paradoxically, we found extensive differences in selective contracting between providers and over time, without any distinguishable effect on provider budgets. Potential explanations include selective contracting having limited impact on patient flows,^51^ with insurers required to (partially) reimburse noncontracted care,^17^ hospitals levering market power to compensate reductions in income,^52^ hospitals shifting costs between insurers as a response to selective contracting,^53^ or insurers using selective contracting to concentrate care symmetrically between providers without affecting total expenditures.^54^ In theory, selective contracting could even steer patients towards high-quality providers without affecting total provider expenditures, for example if prices adjust downwards to compensate increases in the number of patients. Additional studies on the correlation between active purchasing (eg, selective contracting) and quality measures could further elucidate the role of active purchasing in stimulating market efficiency.

###  Strengths and Limitations

 This research provides a new indicator to empirically investigate reallocations of funds between providers as a proxy for active purchasing. However, factors unrelated to purchasing may influence market share changes, such as shifts in financing arrangements and socio-demographic trends. Due to these limitations, MAI should be interpreted as one proxy for active purchasing, acknowledging that external factors may affect MAI. Furthermore, purchasing may constitute actions that do not result in reallocations of funds. For example, active purchasing may focus on across-the-board quality improvements or cost containment without affecting provider expenditures. Recent examples of multi-year fixed budget contracts aiming to improve quality indicate that insurers may use other strategies to improve efficiency.^55-57^ Insurers may also aim to direct active purchasing to concentrate specific types of care and reduce other types of care within a hospital, without affecting the total budget, as is suggested by the high extent of selectively contracting specific procedures in the Netherlands. In theory, concentration efforts could be reciprocal between providers, resulting in improved efficiency without budgetary changes.^54^ This would cause the MAI to underestimate active purchasing. Ideally, detailed data on provider expenditures per location and per payer would allow us to test these alternative explanations, but collecting these detailed data is an area for future research.

 Data constructs may also affect MAI. Data errors unequivocally increase MAI. Contrary, if small providers, which are exempted from the dataset experience higher reallocations, MAI may be underestimated. Especially for municipal care and personal budget care, only data for relatively large, formal care providers is available. If independent contractors and specialised cleaning/household assistance companies, which are missing from the dataset, experience higher reallocations, MAI is biased downwards. Missing providers may also reduce external validity of our explanatory analyses. Potentially, quality and MAI could be correlated for very small providers outside the dataset. However, the results are valid for large providers covering 93% of total spending. Furthermore, internal validity of the results from the explanatory regression analysis seems unaffected by imputation of missing values.

 While the MAI may be used to compare health systems, the optimal or baseline level of reallocations remains undetermined. A certain amount of reallocations are necessary to accommodate demographic changes and improve efficiency, but very high budgetary volatility may risk provider continuity. Furthermore, reallocations do not necessarily imply improved efficiency, ie, being geared towards high quality providers.

###  Policy Implications and Suggestions for Future Research

 Despite theory predicting that managed competition should increase reallocations of funds between providers, evidence finds relatively low reallocations in the Dutch hospital sector. While provider expenditures in the Dutch system are constrained by global budget agreements, users may still have a significant role: if users are inclined to visit the nearest hospital only, there may be little room to reallocate provider expenditures. That would render competitive payer reforms useless in terms of allocative efficiency. However, studies do demonstrate some willingness to travel for care or acceptance of restrictive policies.^49,58,59^ Furthermore, municipal care and personal budgets do show potential for high MAI. Especially in a small, rich, very densely populated country with high quality road infrastructure such as the Netherlands, potential for competitive payer reform to improve allocative efficiency exists. Possibly, implementation of managed competition in the Netherlands is imperfect, for example due to a lack of quality transparency.^17^ Improving valid quality indicators may enable insurers to select well-performing providers.

 Other potential explanations may be more structural: payers may lack legitimacy, bargaining power and purchasing tools to reallocate budgets.^37^ For example, establishing a good relationship with providers may be beneficial for improving outcomes, but may hinder active purchasing.^40,60,61^ Evidence from Germany stipulates frictions due to competition in a corporatist health system.^62^ Lastly, in the United Kingdom active purchasing was found to be incompatible with the mindset of commissioners.^63^ A similar explanation could be applicable to the Netherlands: both insurers and LTC- providers may experience difficulties in deciding which provider patients should visit. On the other hand, evidence from the United States and the Netherlands suggests that purchasers are to a certain extent able and willing to steer patients.^51,64,65^ However, purchasers may be more inclined to retain the status-quo. Boonen and Schut hypothesize the credible commitment problem: if consumers do not trust purchasers to act prudently on their behalf, insurers fear loss of reputation and customers upon selective contracting of providers.^6,66^

 Concentrated purchaser markets may exert market power over providers. While purchaser concentration increased during our study period, reallocations of funds between providers did not. Possibly, increases in purchaser concentration were counteracted by increases in provider concentration. Additional research on purchaser-provider bargaining is required to assess the effect of market concentration on allocative efficiency. Related, reallocations require sufficient potential of providers to increase capacity, or -in case of budgetary reductions- to reduce expenditures, eg, personnel or capital expenditures. The expenditure side might be relatively fixed, so large changes in the budget might risk provider continuity. As insurers are obliged to procure sufficient volumes of care, insufficient flexibility and excess capacity may form a barrier for reallocation of budgets in the hospital market. We find that provider concentration is associated with higher reallocations, consistent with some but not all literature.^30-33^

 Managed competition has been advocated to constrain total costs, although this was not the main objective of the 2007 competitive reform.^20,67^ If insurers pursue cost containment under managed competition uniformly, this could be reflected in a low MAI. However, between 2007 and 2012 hospital expenditures grew rapidly.^20^ In 2012, the government addressed fast growing hospital expenditures by negotiating agreements between hospital and insurer representatives to cap real overall hospital growth to 2.5% per year. These industry agreements were renewed in 2018, with real growth rates gradually declining towards 0% in 2022. These agreements did not formally prevent budget reallocations, as within the total hospital budget, purchasers were still expected to negotiate individual budgets, but the 2.5% limit may have informally functioned as an anchor in individual negotiations. While this could have reduced reallocations, no clear trend break was found after 2012.

 Governments contemplating competitive reforms and managed competition may find these measures insufficient to improve allocative efficiency. Besides improving market preconditions — eg, quality transparency, sufficient provider capacity, adequate antitrust policy —, monitoring and benchmarking purchaser activity could further incentivise improvements in efficiency-driven budget reallocations.^68^ The MAI could be used as one of the indicators for purchaser performance.

## Conclusion

 Contrary to the theory of managed competition, low reallocations of funds between providers were found in the competitive Dutch hospital sector, questioning the premise that managed competition improves allocative efficiency by means of selectively contracting high-quality providers. Competitive reform and managed competition may not be sufficient to ensure static and dynamic efficiency in the healthcare market. Policy-makers may need to monitor progress, remove barriers and adjust incentives to obtain well-functioning healthcare markets. The MAI provides a useful indicator for policy-makers to monitor allocative activity in different purchaser systems.

## Acknowledgements

 The authors would like to acknowledge Raf van Gestel and participants of the LolaHESG conference 2016 for constructive comments on a previous version of this manuscript.

## Ethical issues

 No approval from an ethical committee was required, as the study was conducted on publicly available datasets according to Dutch regulations (WTZa, Wet Toetreding Zorgaanbieders). Dutch legislature exempt ethics approval for studies that use aggregate, pseudonomized data (WMO, Wet Medisch-wetenschappelijk onderzoek met mensen). Use of publicly available company data is permitted for scientific research under Dutch legislation (AGV, algemene verordening gegevensbescherming).

## Competing interests

 Authors declare that they have no competing interests.

## Endnotes

 [1] For example, if a payer reallocates 5% of the market, he takes 5% from providers and gives 5% to other providers. The sum of absolute changes in the market is 10%, but the percentage of the market that has been redistributed is half that (5%). [2] For example, if insurer A contracts provider X for 300 of the 400 potential types of care, the contracting index is 0.75.

## Supplementary files

Supplementary file 1 contains Figures S1-S6 and Tables S1-S9.
