# Supplementary material for: Measuring Active Purchasing in Healthcare: Analysing Reallocations of Funds Between Providers to Evaluate Purchasing Systems Performance in the Netherlands
Source: Int J Health Policy Manag. 2023 Sep 17;12:7506. doi: 10.34172/ijhpm.2023.7506 (PMC10590252; doi:10.34172/ijhpm.2023.7506)
Supplement: Supplementary file 1 — contains Figures S1-S6 and Tables S1-S9. [file ijhpm-12-7506-s001.pdf]

Article title: Measuring Active Purchasing in Healthcare: Analysing Reallocations of Funds Between Providers to Evaluate Purchasing Systems Performance in the Netherlands

Journal name: International Journal of Health Policy and Management (IJHPM)

Authors' information: Niek Waltherus Stadhouders[1]*, Xander Koolman[2], Marit A.C. Tanke[1], Hans Maarse[3], Patrick P.T. Jeurissen[1]

[1]Scientific Institute for Quality of Healthcare (IQ Healthcare), Radboud University Medical Center, Nijmegen, The Netherlands.

[2]School of Business and Economics, Vrije Universiteit, Amsterdam, The Netherlands.

[3]Department of Health Services Research, School for Public Health and Primary Care (Caphri), Faculty of Health, Medicine and Life Sciences, Maastricht University, Maastricht, The Netherlands.

***Correspondence to**: Niek Waltherus Stadhouders, Email: niek.stadhouders@radboudumc.nl

Citation: Stadhouders NW, Koolman X, Tanke MAC, Maarse H, Jeurissen PPT. Measuring active purchasing in healthcare: analysing reallocations of funds between providers to evaluate purchasing systems performance in the Netherlands. Int J Health Policy Manag. 2023;12:7506. doi:10.34172/ijhpm.2023.7506

Supplementary

Table S1: Annual sectoral expenses and completeness of the data.

	Hospital care*		Long term care		Mental care**		Social care		Personal budgets***	
**Year**	**Spending (mln)**	**Completeness**	**Spending (mln)**	**Completeness**	**Spending (mln)**	**Completeness**	**Spending (mln)**	**Completeness**	**Spending (mln)**	**Completeness**
**2006**	€ 15,216	68%	€ 17,758	66%	€ 4,097	84%			€ 1,125	8%
**2007**	€ 14,792	91%	€ 17,025	93%	€ 4,170	102%	€ 1,411	0%	€ 1,333	10%
**2008**	€ 15,875	89%	€ 18,063	98%	€ 4,737	99%	€ 1,475	71%	€ 1,661	10%
**2009**	€ 16,788	90%	€ 19,728	96%	€ 5,151	99%	€ 1,533	77%	€ 1,958	11%
**2010**	€ 17,708	93%	€ 20,295	96%	€ 5,143	103%	€ 1,541	81%	€ 2,158	12%
**2011**	€ 18,274	90%	€ 21,261	96%	€ 5,629	100%	€ 1,456	91%	€ 2,244	12%
**2012**	€ 19,661	87%	€ 23,500	94%	€ 5,626	101%	€ 1,511	91%	€ 2,527	12%
**2013**	€ 21,726	85%	€ 23,468	94%	€ 5,838	89%	€ 1,561	80%	€ 2,415	12%
**2014**	€ 22,878	84%	€ 23,798	93%	€ 5,674	80%	€ 1,714	64%	€ 2,411	13%
**2015**	€ 22,324	98%	€ 18,057	93%	€ 4,071	68%	€ 4,943	64%	€ 1,274	26%
**2016**	€ 23,407	97%	€ 17,715	96%	€ 4,148	87%	€ 4,945	70%	€ 1,568	20%
**2017**	€ 24,202	96%	€ 18,086	97%	€ 4,069	85%	€ 4,899	62%	€ 1,753	0%
**2018**	€ 24,935	97%	€ 19,043	97%	€ 4,189	85%	€ 5,111	59%	€ 1,919	0%
**2019**	€ 26,002	97%	€ 21,087	94%	€ 4,605	79%	€ 5,111	65%	€ 2,087	0%

Source: Ministry of Health, Welfare and Sports annual accounts, 2006-2019; Annual reports database (DigiMV 2007-2019)
***up to 2015 independent medical specialist reimbursements were excluded from the dataset**
****excess of 100% may occur due to exclusion of housing costs from the sector macro-budget, which are included in provider expenses**
*****from 2017 personal budgets cannot be distinguished as a separate category in the dataset**

Table S2: Descriptive pooled statistics, 2007-2019				
**Variable**	**Mean**	**SD**	**Maximum**	**N**
**Hospital budget**	€77,200,000	€135,000,000	€1,120,000,000	3,344
**% ITC**	0.49	0.50		3,342
**Elderly care budget**	€26,000,000	€41,500,000	€508,000,000	6,427
**% home care for elderly**	0.69	0.36		6,198
**Disability care budget**	€36,700,000	€65,000,000	€734,000,000	2,522
**% home care for disabled**	0.45	0.42		2,344
**Mental care budget**	€22,800,000	€58,400,000	€692,000,000	2,671
**Social care budget**	€3,237,685	€ 9,024,436	€217,000,000	7,576
**Personal budgets spent at providers**	€522,458	€ 1,049,869	€15,900,000	5,205
**Total provider budget**	€39,000,000	€103,000,000	€1,870,000,000	20,319
**Capital expenditures ratio**	0.10	0.09	0.36*	16,723
Source: Annual reports database (DigiMV 2007-2019). Note: all minimum values are zero. *99% of the distribution.				

Table S3: descriptive statistics for the main regression analysis (2015-2019)					
**Variable**	N	Mean	SD	Min	Max
**Market share in hospital market (%)**	942	0.48%	0. 73	0.00%	4.41%
**Delta market share (%points)**	919	0.01	0.01	-0.50	0.74
**Absolute Delta market share (%points)**	919	0.03	0.01	0.00	0.74
**Average contracting index (ACI)**	942	0.77	0.26	0.0028	1.00
**Delta ACI**	698	0.02	0.14	-0.84	0.77
**Absolute delta ACI**	698	0.08	0.12	0.00	0.84
**Percentage fixed costs**	714	0.07	0.07	0.00	1
**ITC dummy**	942	0.51	0.50	0	1
**Mean z-score structural quality**	494	-0.05	0.33	-2.20	1.44
**Mean z-score process quality**	483	0.00	0.88	-7.72	6.71
**Mean z-score outcome quality**	327	-0.01	0.41	-2.68	1.71
**Hospital standardized mortality ratio (hsmr)**	279	98.54	10.90	67	125
Source: Annual reports database (DigiMV 2007-2019), Quality of care database (Zorginzicht 2010-2018), HSMR database (DHD 2010-2014), Mediquest database (2015-2019). Note: all minimum values are zero unless listed otherwise. **note: mean z-scores over a set of indicators do not necessarily retain a standard-normal distribution.					

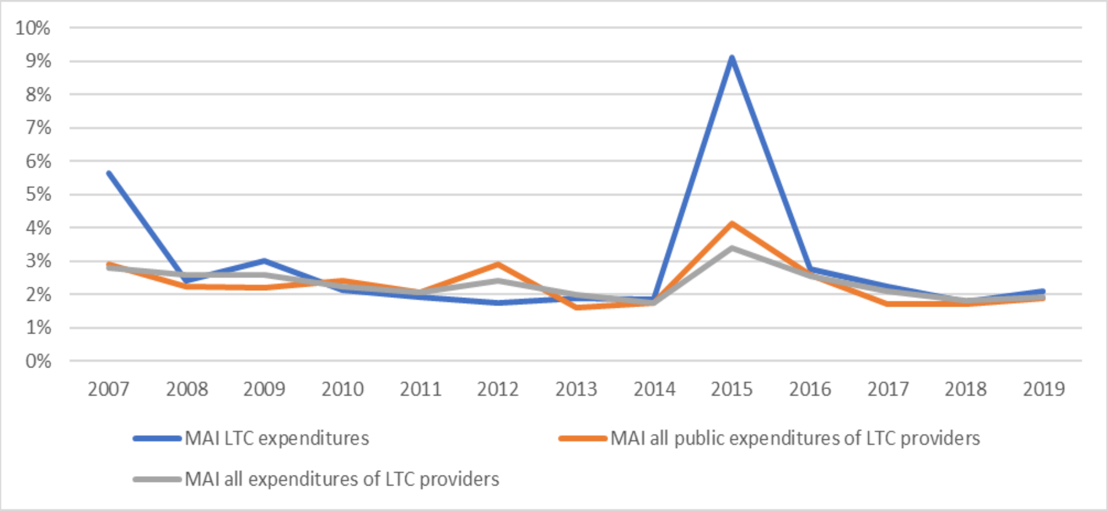

Figure S1: MAI of LTC providers, corrected for administrative budget reallocations, 2006/7-2018/9

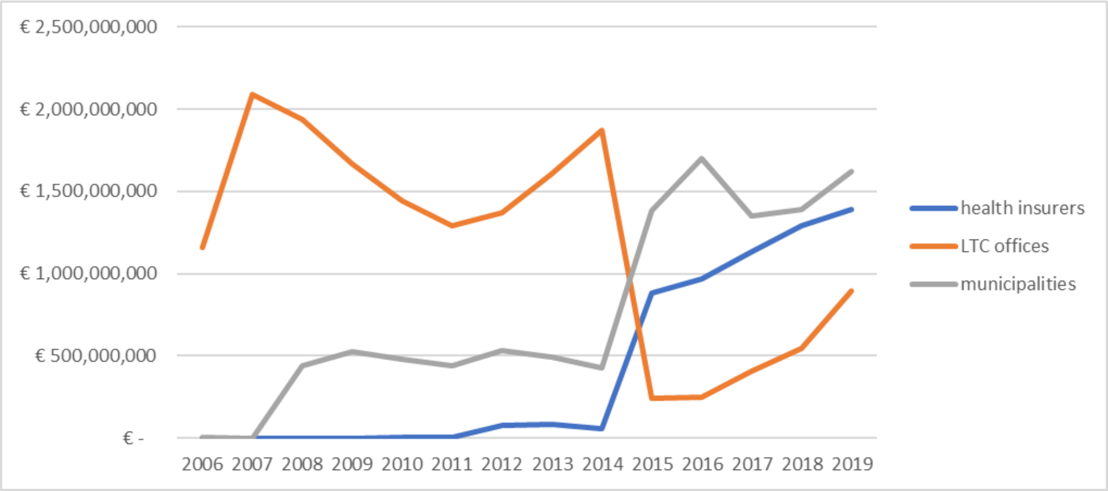

Figure S2: expenditures of home care providers in different sectors

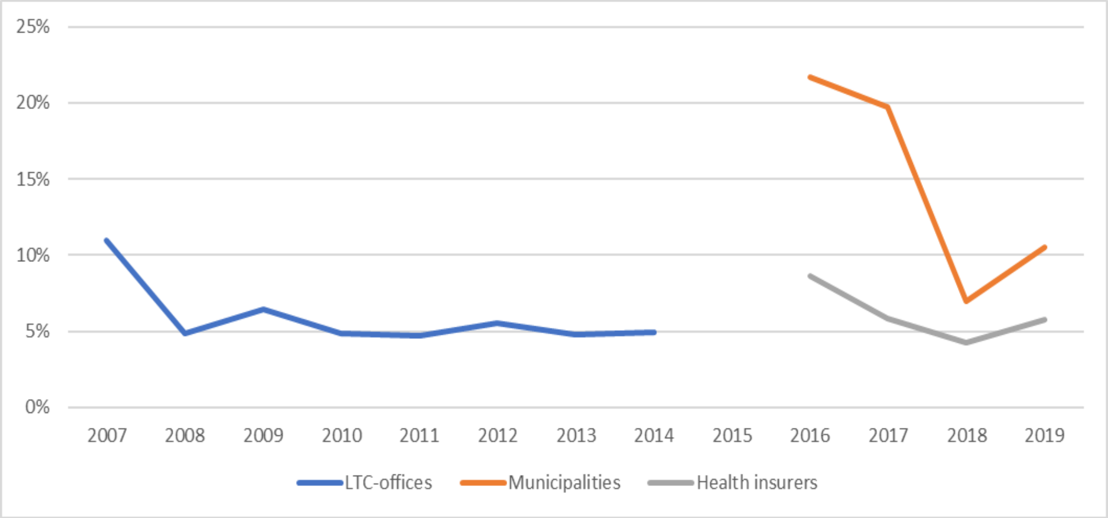

Figure S3: MAI of home care providers in different sectors, 2007/8 to 2018/19

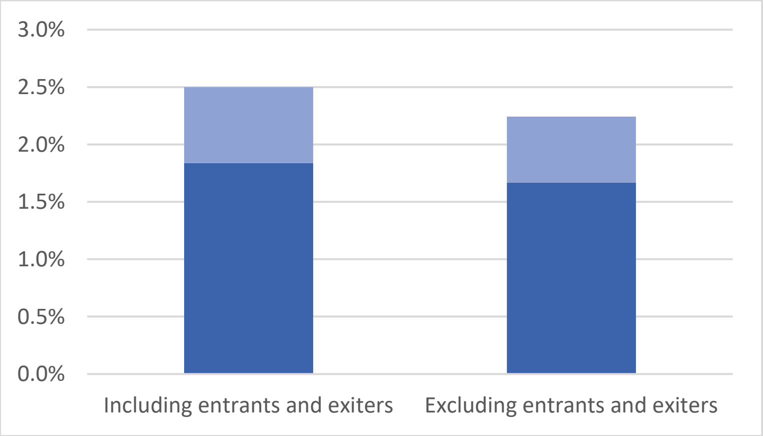

Figure S4: MAI of all care, effects of excluding entry and exit of providers. Structural trend is calculated as differences in market share over a period of two years.

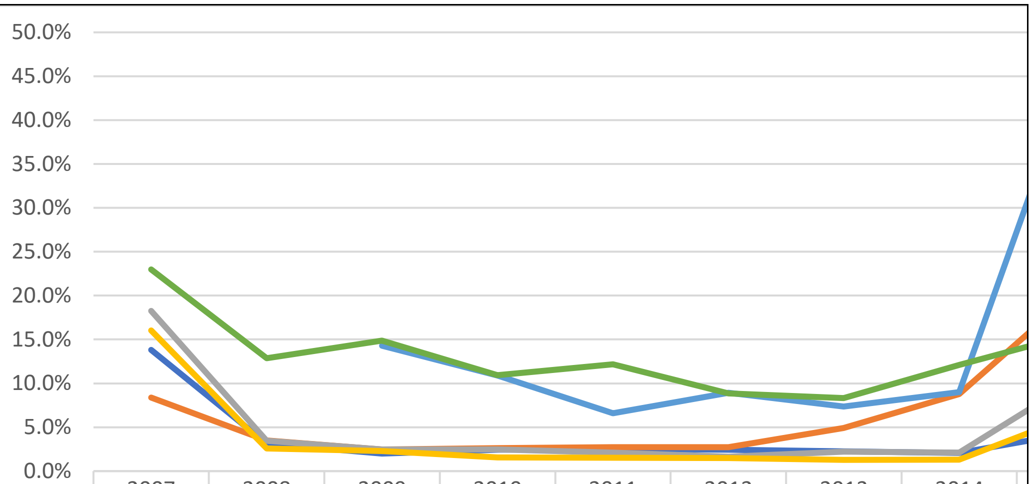

	2007	2008	2009	2010	2011	2012	2013	2014
Hospital care	13.8%	3.1%	2.0%	2.4%	2.5%	2.5%	2.2%	2.1%
Mental care	8.4%	3.5%	2.5%	2.6%	2.7%	2.7%	4.9%	8.8%
Elderly care	18.3%	3.5%	2.5%	2.5%	2.1%	1.6%	2.2%	2.1%
Disability care	16.0%	2.6%	2.3%	1.6%	1.5%	1.5%	1.3%	1.3%
Municipal care			14.3%	10.9%	6.6%	8.9%	7.4%	9.0%
Personal budgets	23.0%	12.9%	14.9%	10.9%	12.2%	8.9%	8.3%	12.1%

Hospital care Mental care Elderly care Disability care Munic

Figure S5: robustness check including market share reallocations between non-missing providers

Table S4: main regression results (pooled linear regression with clustered standard errors and year fixed-effects) including imputed data				
	**Relative market share changes**	**Absolute market share changes**	**Relative market share changes, including HSMR**	**Absolute market share changes, including HSMR**
**Delta ACI**	-0.0003 (0.005)	0.0002 (0.004)	-0.03 (0.06)	0.03 (0.05)
**MS (t-1)**	-1.65 (0.46***)	2.68 (0.69***)	0.18 (0.93)	2.13 (0.69**)
**MS^2 (t-1)**	33.96 (14.92*)	-24.877 (19.135)	-3.89 (26.14)	-21.59 (13.19)
**Fixed costs (t-1)**	0.002 (0.01)	-0.013 (0.006*)	-0.09 (0.13)	0.01 (0.13)
**ITC dummy**	-0.002 (0.003)	-0.0002 (0.004)	0.01 (0.01)	-0.01 (0.01)
**Structural Q (t-1)**	-0.001 (0.002)	-0.001 (0.002)	-0.01 (0.02)	-0.01 (0.01)
**Process Q (t-1)**	-0.0002 (0.001)	-0.0001 (0.001)	-0.01 (0.01)	-0.001 (0.01)
**Outcome Q (t-1)**	0.0003 (0.002)	0.002 (0.002)	0.02 (0.01)	0.01 (0.02)
**HSMR (t-1)**	0	0	-0.0002 (0.0002)	0 (0)
**Year=2016**	Baseline	Baseline		0
**Year=2017**	0.003 (0.003)	-0.003 (0.002)	Baseline	Baseline
**Year=2018**	0.01 (0.003***)	-0.002 (0.003)	0.02 (0.01**)	-0.01 (0.01)
**Year=2019**	0.01 (0.003**)	-0.004 (0.002)	0.02 (0.01***)	-0.01 (0.004)
**Constant**	-0.003 (0.003)	0.01 (0.004)	0.01 (0.02)	0.02 (0.02)
**# observations**	1059	1059	208	208
**F-statistic**	3.95	11.59	2.34	3.29
Note: ACI=average contracting index; MS=market share; HSMR=hospital standardized mortality ratio; # = omitted due to collinearity (all observations of HSMR have ITC=0). Standard errors are in parentheses. *=5% significance, **=1% significance, ***=0.1% significance.				

Table S5: Robustness checks on contracting index (pooled linear regression with clustered standard errors and year fixed-effects)		
	**RMSC**	**AMSC**
**ITC**	-0.01 (0.01)	0.004 (0.01)
**UMC**	0.03 (0.01)	0.002 (0.01)
**Market share in t-1**	-2.47** (0.77)	3.05** (1.12)
**MS^2 in t-1**	32.35(19.56)	-34.84 (26.56)
**ACI of Hospitals**	-0.01 (0.01)	0.01 (0.01)
**ACI of ITC**	0.02* (0.01)	-0.02 (0.01)
**ACI of UMC**	-0.11 (0.20)	-0.05 (0.10)
**N**	689	689
**F**	6.06	51.42
**R^2**	0.08	0.21
Notes: RMSC=relative market share changes; AMSC=absolute market share changes; hospitals are used as baseline. Year dummies and constants are suppressed for brevity. sign. *<5%;**<1%;***<0.1%		

Table S6: robustness checks on size effects and fixed costs effects (pooled linear regression with clustered standard errors and year fixed-effects)						
	**Hospital care**		**Elderly care**		**Disability care**	
	**RMSC**	**AMSC**	**RMSC**	**AMSC**	**RMSC**	**AMSC**
**MS in t-1**	0.89** (0.33)	5.28*** (0.40)	0.07 (0.81)	4.04*** (0.59)	-0.33 (0.49)	5.04*** (0.38)

**MS² in t-1**	-39.86* (8.71)	-42.58* (19.46)	-335.11** (110.81)	129.24** (9.42)	99.22*** (18.66)	-60.18*** (15.03)
**FC**	-0.01 (0.01)	0.002 (0.01)	0.001 (0.003)	-0.02* (0.01)	0.002 (0.01)	-0.01 (0.01)
**N**	2,687	2,687	5,298	5,298	1,992	1,992
**F**	8.31	64.22	12.92	34.15	23.43	53.39
**R²**	0.12	0.34	0.10	0.22	0.11	0.27
	**Mental care**		**Municipal care**		**Personal budgets**	
	**RMSC**	**AMSC**	**RMSC**	**AMSC**	**RMSC**	**AMSC**
**MS in t-1**	-2.72 (1.74)	7.20*** (1.01)	-5.61 (5.17)	14.31** (4.55)	2.58 (3.10)	24.74*** (2.53)
**MS² in t-1**	25.51* (12.51)	-14.672 (7.51)	-253.71 (12.22)	164.78 (286.01)	-1699.19* (716.08)	-1466.54** (672.09)
**FC**	-0.04 (0.04)	0.07 (0.04)	-0.04 (0.03)	-0.03 (0.02)	-0.001 (0.01)	-0.01* (0.01)
**N**	1,819	1,819	5,097	5,097	3,674	3,674
**F**	27.16	930.39	13.17	67.82	16.79	39.82
**R²**	0.04	0.25	0.12	0.30	0.15	0.33

Notes: RMSC=relative market share changes; AMSC=absolute market share changes. Year dummies and constants are suppressed for brevity. sign. *<5%;**<1%;***<0.1%

Table S7: robustness checks on size effects, outpatient care and fixed costs effects (pooled linear regression with clustered standard errors and year fixed-effects)

	**Hospital care**		**Elderly care**		**Disability care**	
	**RMSC**	**AMSC**	**RMSC**	**AMSC**	**RMSC**	**AMSC**
**MS in t-1**	0.90* (0.41)	4.95*** (0.49)	-0.06 (0.80)	4.15*** (0.61)	-0.33 (0.50)	5.03*** (0.38)
**MS² in t-1**	-39.97* (20.05)	-33.78 (19.24)	-321.88** (108.52)	118.76** (47.81)	98.50*** (19.04)	-59.93*** (15.07)
**FC**	-0.01 (0.01)	-0.001 (0.01)	-0.01** (0.003)	-0.01* (0.01)	0.0004 (0.01)	-0.01 (0.01)
**Outpatient ratio**	0.0005 (0.002)	-0.004 (0.003)	-0.004*** (0.001)	0.004* (0.002)	-0.01** (0.002)	-0.001 (0.001)
**N**	2503	2503	5234	5234	1945	1945
**F**	8.02	66.34	13.6	35.62	22.4	52.34
**R²**	0.13	0.34	0.10	0.22	0.11	0.27

Notes: RMSC=relative market share changes; AMSC=absolute market share changes. Year dummies and constants are suppressed for brevity. sign. *<5%;**<1%;***<0.1%

Table S8: robustness checks on size and fixed costs (pooled linear regression with clustered standard errors and year fixed-effects)

	**Hospital care**		**Elderly care**		**Disability care**	
	**RMSC**	**AMSC**	**RMSC**	**AMSC**	**RMSC**	**AMSC**
**MS in t-1**	1.02 (0.88)	4.64*** (0.46)	-0.10 (0.79)	4.18*** (0.60)	-0.35 (0.49)	5.01*** (0.38)
**MS² in t-1**	-61.16 (46.75)	-8.30 (14.16)	-320.10** (107.56)	115.78* (47.16)	98.88*** (18.66)	-59.08*** (15.05)
**Outpatient ratio**	-0.001 (0.003)	-0.003 (0.003)	-0.004*** (0.001)	0.005* (0.002)	-0.01** (0.002)	-0.001 (0.002)
**N**	2920	2920	5348	5348	1945	1994
**F**	8.84	108.76	15.13	38.01	22.4	55.49
**R²**	0.11	0.34	0.10	0.22	0.11	0.28

Notes: RMSC=relative market share changes; AMSC=absolute market share changes. Year dummies and constants are suppressed for brevity. sign. *<5%;**<1%;***<0.1%

Table S9: Sensitivity analysis excluding contracting data (pooled linear regression with clustered standard errors and year fixed-effects)

	RMSC	AMSC	PMS
**Market share in t-1**	-1.37 (1.09)	4.23** (1.41)	
**MS^2 in t-1**	9.50 (25.86)	-55.99 (31.73)	
**Percentage fixed costs in t-1**	-0.32* (0.12)	0.18 (0.11)	
**structural quality in t-1**	-0.05* (0.02)	-0.003 (0.02)	0.01** (0.004)
**process quality in t-1**	-0.02 (0.02)	0.02 (0.01)	-0.01** (0.003)
**outcome quality in t-1**	0.002 (0.01)	0.003 (0.01)	-0.004** (0.001)
**HSMR**	0.0003 (0.0002)	-0.0004 (0.0002)	0.0002** (0.0001)
**N**	385	385	402
**F**	8.99	10.46	8.71
**R^2**	0.17	0.18	0.19
Notes: RMSC=relative market share changes; AMSC=absolute market share changes; PMS=pooled market shares; Year dummies are suppressed for brevity. sign. *<5%;**<1%;***<0.1%			

Figure S6: MAI of inpatient and outpatient care in LTC

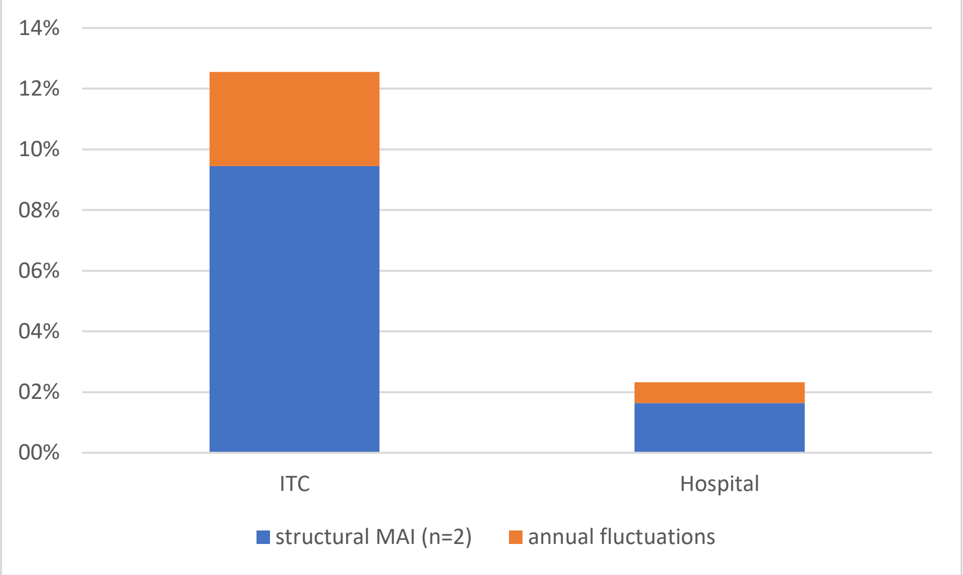

6a: MAI of Independent treatment centers (ITC) and hospitals. Structural trend is calculated as differences in market share over a period of two years.

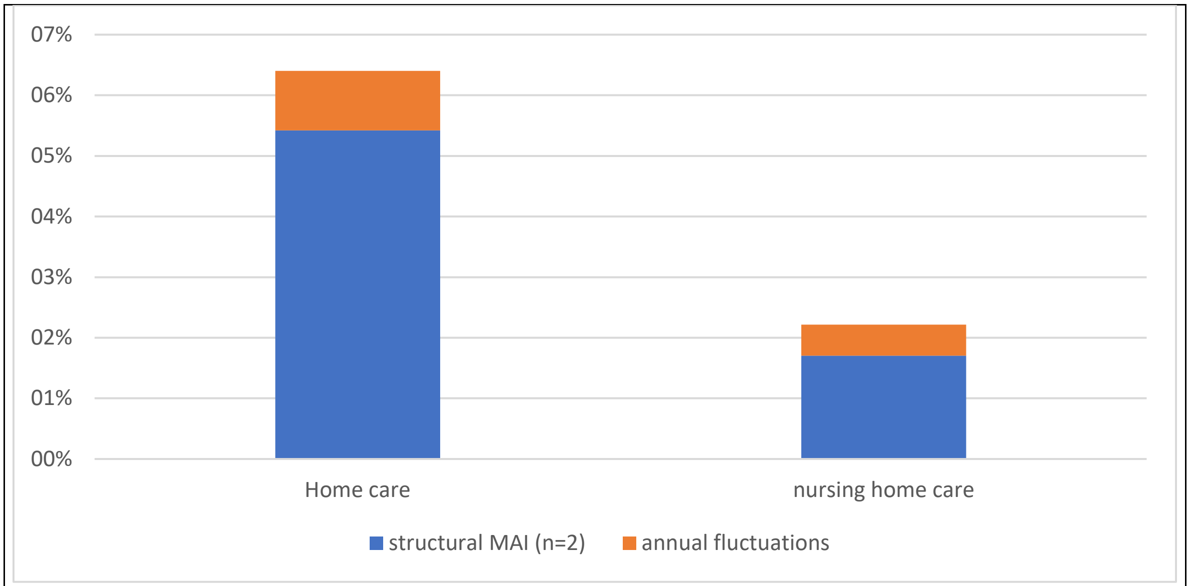

6b) MAI of predominantly outpatient (home care) and predominantly in-patient (nursing home care) elderly care providers. Structural trend is calculated as differences in market share over a period of two years.

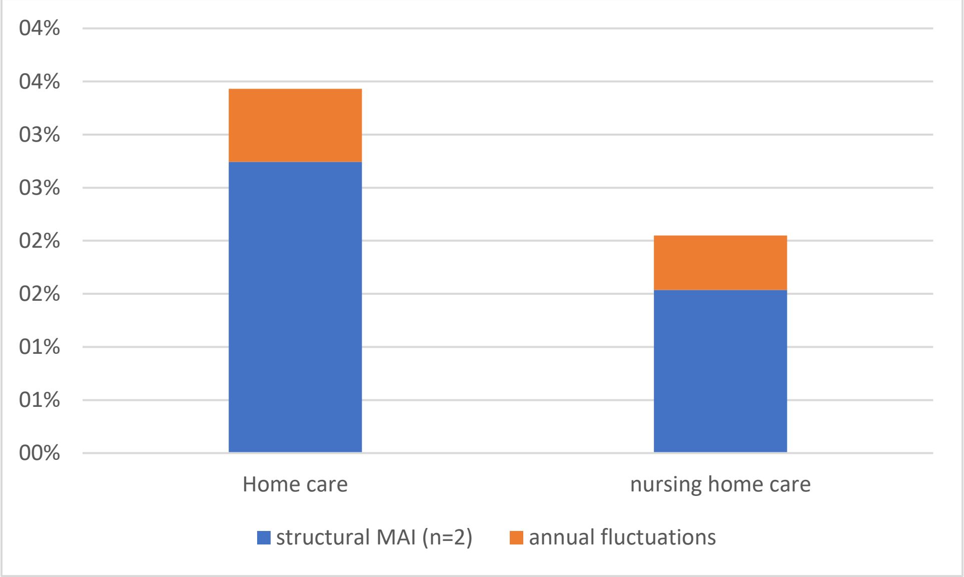

6c) MAI of predominantly outpatient (home care) and predominantly in-patient (nursing home care) disability care providers. Structural trend is calculated as differences in market share over a period of two years.
